# Effects of Dose Reduction or Discontinuation of Benzodiazepine Hypnotics on Sleep and Anxiety in Patients With Insomnia After Long-Term Use

**DOI:** 10.7759/cureus.77936

**Published:** 2025-01-24

**Authors:** Hiroki Endo, Yuki Shigetsura, Kosuke Tsurumi, Naoko Sugita, Shunsaku Nakagawa, Satoshi Imai, Tomohiro Terada

**Affiliations:** 1 Department of Clinical Pharmacology and Therapeutics, Kyoto University Hospital, Kyoto, JPN; 2 Department of Psychiatry, Graduate School of Medicine, Kyoto University, Kyoto, JPN; 3 Department of Medical Neuropharmacology, School of Pharmaceutical Sciences, Wakayama Medical University, Wakayama, JPN

**Keywords:** benzodiazepine receptor agonist, hypnotic, insomnia, long-term use, sleep quality

## Abstract

Background: Long-term prescribing of benzodiazepine receptor agonists (BZRAs) is a problem worldwide, but there are no detailed reports on the effects or side effects of reducing or discontinuing the dose. We retrospectively investigated the efficacy and safety of discontinuing or reducing the dose of BZRAs hypnotics after long-term use.

Methods: Between April 2018 and May 2019, patients with insomnia after long-term use of BZRA hypnotics had BZRA hypnotics discontinued or their dose reduced, and their sleep conditions and anxiety symptoms were assessed. Insomnia severity (primary endpoint) was assessed as the change from baseline after discontinuation or reduction of BZRA hypnotics using the Insomnia Severity Index Japanese version (ISI-J). Changes from baseline in sleep quality and anxiety symptoms (secondary endpoint) from baseline were assessed using the Pittsburgh Sleep Quality Index Japanese version (PSQI-J) and Generalized Anxiety Disorder-7 (GAD-7), respectively. The adverse events were recorded during the study period. Statistical analysis was performed using the Wilcoxon matched-pairs signed rank test for changes in BZRA and concomitant medication dose, PSQI-J and GAD-7, and the paired *t*-test for changes in ISI-J.

Results: The changes in ISI-J (discontinuation group: p = 0.07, reduction group: p = 0.91), PSQI-J (discontinuation group: p = 0.19, reduction group: p = 0.19), and GAD-7 scores (discontinuation group, p = 0.27; reduction group, p = 0.81) were not significant after the BZRA hypnotics were discontinued or reduced. Concomitant medications (antipsychotics and antidepressants) did not change from the baseline. The incidence of each adverse event did not change during the study period.

Conclusion: BZRA hypnotics can be discontinued or their dose reduced after long-term use without worsening sleep conditions and anxiety symptoms. Our results may provide a basis for the safe and effective discontinuation or dose reduction of long-term benzodiazepine hypnotics use.

## Introduction

Benzodiazepine receptor agonists (BZRAs) are widely used for the treatment of insomnia and anxiety symptoms [1-3]. BZRAs bind specifically to the gamma-aminobutyric acid (GABA) type A receptor site and increase the affinity of GABA for the receptor and the opening frequency of chloride ion channels, resulting in sleep induction and anxiolytic, anticonvulsant, and muscle relaxant effects [4]. However, because the GABAergic nervous system affects several inhibitory nerves that project to various areas of the brain, BZRAs have several side effects [2]. The typical adverse reactions of BZRAs used in the short term are drowsiness, lethargy, hangover effects, reduced concentration, and attention [5]. Adverse reactions to BZRAs over the long term include cognitive impairment [6], and dependence accompanied by craving, tolerance formation [7, 8] and withdrawal symptoms [9]. Moreover, higher doses of BZRAs and longer durations of use have been associated with higher risks of adverse events [10].

According to a United Nations International Narcotics Control Board report in 2016 [11], BZRAs were more frequently used as sedative hypnotics in Japan than in other countries. The frequent use of BZRAs in Japan is evident in a study using the most recent Nationwide Japanese Claims Database, which showed more than 70% of all hypnotic prescriptions [12]. The high consumption levels observed in Japan may also reflect inappropriate prescription patterns and the associated abuse. In a retrospective cohort study in Japan, 11.8% of new users of BZRAs were consecutively prescribed BZRAs for ≥12 months [13]. BZRAs are still commonly used over the long term in Japan, and this usually necessitates their discontinuation, dose reductions, or replacement with other non-BZRA hypnotics. However, there are few reports on the dose reduction of BZRAs or a switch to other drugs in patients who have used them for longer than 12 months, and patients in clinical practice often hesitate to reduce the dose of BZRAs over the long term. In addition, there are few reports evaluating the effects on sleep and anxiety symptoms, among other things, associated with BZRA dose reduction or discontinuation, and there are no reports of studies examining subjective sleep using rating scales. These data would be useful when reducing or discontinuing BZRA in real-world clinical practice.

To address unnecessary psychotropic polypharmacy, the Japanese Ministry of Health, Labour and Welfare has implemented a policy aimed at reducing health care fees since 2012 [14]. In 2018, the health care fee system was revised to include an additional medical fee for instructing pharmacists or nurses to confirm a change in condition with a change in prescription details when a BZRA administered for 12 months or longer is reduced in dose [15]. At the Department of Psychiatry, Kyoto University Hospital, pharmacists and nurses evaluate changes in sleep and anxiety status using objective measures as part of routine medical care at the time of dose reduction, discontinuation, or dose change of long-term administered BZRA, based on physician orders.

In this study, patients who had been taking BZRA hypnotics for a long duration were retrospectively assessed using electronic medical records for changes in their sleep conditions and anxiety symptoms after dose reduction or discontinuation.

## Materials and methods

Study participants and outcome measurements

This retrospective cohort study included 13 patients with long-term (≥12 months) use of benzodiazepine hypnotics who reported to the Department of Psychiatry, Kyoto University Hospital between April 2018 and May 2019. The necessary patient information was collected retrospectively from electronic medical records. The exclusion criteria included patients with irregular sleep or missing anxiety scores recorded and those for whom a prescription history could not be traced for 12 months prior to the reduction or discontinuation of benzodiazepine hypnotics. Dose reduction or discontinuation of benzodiazepine hypnotics was performed carefully at the psychiatrist’s discretion, without specific protocol, due to the retrospective nature of the study. The benzodiazepine hypnotics were discontinued (n=7) or their doses were reduced (n=6); sleep conditions, anxiety symptoms, and adverse events were assessed afterwards. The patients were retrospectively evaluated for their use of all BZRAs (hypnotics and anxiolytics), sleep status, anxiety symptoms, concomitant medications, and adverse events. Data were collected at baseline (Day 0, when the benzodiazepine hypnotics were discontinued or their doses were reduced) and at follow-up (two to four weeks after baseline) after benzodiazepines were discontinued or the dose was reduced. The maintenance of the changes in the use of the benzodiazepine hypnotics for three months after discontinuing or reducing the dose was also assessed.

Drug usage

The benzodiazepine hypnotics and anxiolytics were converted to diazepam-equivalent doses for comparison. Concomitant medications (antipsychotics and antidepressants) affecting sleep status were also investigated. For the concomitant medications, we used chlorpromazine-equivalent doses for antipsychotics and imipramine-equivalent doses for antidepressants [16] for comparison. The diazepam-equivalent doses (sum of hypnotic and anxiolytic doses), chlorpromazine-equivalent doses, and imipramine-equivalent doses were compared at baseline and during follow-up.

Assessment of efficacy and safety

Efficacy was investigated retrospectively using scores assessed by a self-report questionnaire. The Japanese version of the Insomnia Severity Index (ISI-J), validated for use with Japanese patients, was used to assess insomnia severity [17], and the scores at baseline and during follow-up (two to four weeks later) were used as the primary efficiency measure. The ISI-J is a seven-item self-reported questionnaire for assessing the severity of sleep onset, sleep maintenance, early morning awakening problems, sleep dissatisfaction, interference of sleep difficulties with daytime functioning, noticeability of sleep problems by others, and distress caused by sleep difficulties. Each item is rated on a five-point Likert scale (0 = no problem; 4 = very severe problem), and the total score ranges from 0 to 28. Higher scores denote worse insomnia (with 0 ≤ score ≤ 7 = no clinically significant insomnia; 7 < score ≤ 14 = subthreshold insomnia; 14 < score ≤ 21 = moderate severity clinical insomnia; and 21 < score ≤ 28 = severity clinical insomnia.).

The Japanese version of the Pittsburgh Sleep Quality Index (PSQI-J) for subjective sleep quality [18, 19] and Generalized Anxiety Disorder 7-Item (GAD-7) [20] scores, validated measures in Japanese patients, were compared at baseline and follow-up as secondary efficiency measures. The PSQI-J assesses seven components: sleep quality, sleep latency, sleep duration, habitual sleep efficiency, sleep disturbances, use of sleep medicine, and daytime dysfunction. Each item is rated on a scale of 0 to 3 (0 = no insomnia; 3 = severe insomnia), and the total score ranges from 0 to 21. Total PSQI-J scores of >5.5 suggest clinical insomnia (higher, worse). The GAD-7 comprises seven items and reflects generalized anxiety symptoms during the two weeks preceding the assessment. Each item is rated on a four-point Likert scale (0 = not at all; 3 = problem nearly every day), and the total score ranges from 0 to 21 (0 ≤ score < 5 = minimal anxiety, 5 ≤ score < 10 = mild anxiety, 10 ≤ score < 15 = moderate anxiety, 15 ≤ score ≤ 21 = severe anxiety).

Safety was assessed by evaluating adverse events throughout the study. Any new or worsening signs and symptoms of illness, regardless of their relationship with the changes in medication, were recorded as adverse events.

Statistical analysis

General Overview

Statistical analyses were performed using GraphPad Prism Version 7 (GraphPad Software Inc., San Diego, CA, USA). Continuous variables were analyzed using the Shapiro-Wilk test to test for normal distribution. Statistical significance was set at a two-sided p-value of < 0.05.

Drug Usage Analysis

Changes in the use of benzodiazepines (diazepam equivalents) and concomitant medications (chlorpromazine and imipramine equivalents) at baseline and at follow-up were statistically examined using the Wilcoxon matched-pair signed-rank test.

Primary and Secondary Efficiency Analysis

Changes in insomnia severity at baseline and during follow-up were statistically examined. Comparisons of insomnia severity (mean ISI-J total score) were performed using corresponding t-tests. The comparison of the changes in sleep quality (PSQI-J total score and seven component scores) and anxiety symptoms (GAD-7 total score) at baseline and during follow-up was performed using the Wilcoxon matched-pairs signed rank test.

Safety Analysis

Safety was statistically examined based on the frequency of adverse events. The incidence of adverse events at baseline and during the study were compared using Fisher’s exact test because 20% of the cells had expected frequencies of < 5.

## Results

Patient summary

A summary of the medicines used by the patients and various scores is presented in Table 1.

**Table 1:** Patient Summary

Patient No.	Group	Discontinued/Reduced benzodiazepine hypnotics	The duration from baseline to follow-up	Benzodiazepines used at baseline (total diazepam equivalent dose)	Benzodiazepines used at follow-up (total diazepam equivalent dose)	Non-benzodiazepine hypnotics used at baseline	Non-benzodiazepine hypnotics used at follow-up	Antidepressants taken concomitantly at baseline (total imipramine equivalent dose)	Antidepressants taken concomitantly at follow-up (total imipramine equivalent dose)	Antipsychotics taken concomitantly at baseline (total chlorpromazine equivalent dose)	Antipsychotics taken concomitantly at follow-up (total chlorpromazine equivalent dose)	ISI-J score baseline	ISI-J score follow-up	PSQI-J score baseline	PSQI-J score follow-up	GAD-7 score baseline	GAD-7 score follow-up
1	Discontinuation	Flunitrazepam	4 weeks	Flunitrazepam 1 mg, alprazolam 0.8mg (10 mg)	Alprazolam 0.8 mg (5 mg)	None	None	Duloxetine 30 mg, trazodone 25 mg, escitalopram 10 mg (287.5 mg)	Duloxetine 30 mg, trazodone 25 mg, escitalopram 10 mg (287.5 mg)	Quetiapine 25 mg (37.9 mg)	Quetiapine 25 mg (37.9 mg)	10	10	9	11	4	2
2	Discontinuation	Brotizolam	4 weeks	Brotizolam 0.25 mg, etizolam 1.5 mg (10 mg)	Etizolam 1.5 mg (5 mg)	None	None	Amitriptyline 10mg (10 mg)	Amitriptyline 10mg (10 mg)	None (0 mg)	None (0 mg)	11	7	7	6	5	7
3	Discontinuation	Nitrazepam	4 weeks	Nitrazepam 5 mg (5 mg)	None (0 mg)	Suvorexant 20 mg, ramelteon 8 mg	Suvorexant 20 mg, ramelteon 8 mg	Sertraline 100 mg, mirtazapine 45 mg (375 mg)	Sertraline 100 mg, mirtazapine 45 mg (375 mg)	Levomepromazine 10 mg (10 mg)	Levomepromazine 10 mg (10 mg)	18	10	13	12	18	8
4	Discontinuation	Brotizolam	4 weeks	Brotizolam 0.25 mg, lorazepam 1.5 mg (11.25 mg)	Lorazepam 1.5 mg (6.25 mg)	None	None	Trazodone 100 mg (50 mg)	Trazodone 150 mg (75 mg)	Haloperidol 9 mg (450 mg)	Haloperidol 12 mg (600 mg)	16	2	11	3	13	1
5	Discontinuation	Brotizolam	4 weeks	Brotizolam 0.25 mg ethyl loflazepate 2 mg, etizolam 1 mg (14 mg)	Ethyl loflazepate 2 mg, etizolam 1 mg (9 mg)	None	None	Sertraline 25 mg (37.5 mg)	Sertraline 25 mg (37.5 mg)	None (0 mg)	None (0 mg)	9	8	9	7	5	4
6	Discontinuation	Brotizolam	4 weeks	Brotizolam 0.125 mg bromazepam 2 mg (6.25 mg)	Bromazepam 2 mg (4 mg)	None	None	None (0 mg)	None (0 mg)	Quetiapine 25 mg (37.9 mg)	Quetiapine 25 mg (37.9 mg)	8	5	6	6	5	4
7	Discontinuation	Brotizolam	4 weeks	Brotizolam 0.25 mg (5 mg)	None (0 mg)	Ramelteon 8 mg	Ramelteon 8 mg	Trazodone 100 mg (50 mg)	Trazodone 100 mg (50 mg)	Aripiprazole 12 mg, quetiapine 100 mg (451.5 mg)	Aripiprazole 12 mg, quetiapine 100 mg (451.5 mg)	12	12	16	13	7	8
8	Reduction	Flunitrazepam	4 weeks	Flunitrazepam 2 mg, diazepam 5 mg (15 mg)	Flunitrazepam 1 mg, diazepam 5 mg (10 mg)	None	None	None (0 mg)	None (0 mg)	Risperidone 7 mg (700 mg)	Risperidone 7 mg (700 mg)	5	1	6	7	0	3
9	Reduction	Flunitrazepam	2 weeks	Flunitrazepam 1mg (5 mg)	Flunitrazepam 0.5 mg (2.5 mg)	None	Suvorexant 20 mg	None (0 mg)	None (0 mg)	Aripiprazole LAI 400 mg (400 mg)	Aripiprazole LAI 400 mg (400 mg)	3	16	12	17	3	3
10	Reduction	Flunitrazepam	2 weeks	Flunitrazepam 1 mg (5mg)	Flunitrazepam 0.25 mg (2.5 mg)	None	None	none (0mg)	None (0mg)	Olanzapine 10 mg (400 mg)	Olanzapine 15 mg (600 mg)	2	4	9	7	2	6
11	Reduction	Flunitrazepam	3 weeks	Flunitrazepam 2 mg (10 mg)	Flunitrazepam 1 mg (5 mg)	None	None	Mirtazapine 15 mg (75 mg)	Mirtazapine 15 mg (75 mg)	Aripiprazole 3 mg, quetiapine 12.5 mg (93.9 mg)	Aripiprazole 3 mg, quetiapine 12.5 mg (93.9 mg)	12	11	11	9	12	3
12	Reduction	Flunitrazepam	4 weeks	Flunitrazepam 4 mg brotizolam 0.25 mg (25 mg)	Flunitrazepam 1 mg brotizolam 0.25 mg (10 mg)	None	None	None (0 mg)	None (0 mg)	Olanzapine 15 mg, levomepromazine 10 mg (610 mg)	Olanzapine 15 mg, levomepromazine 10 mg (610 mg)	11	8	12	12	3	5
13	Reduction	Flunitrazepam	4 weeks	Flunitrazepam 4 mg, brotizolam 0.25 mg, diazepam 10 mg, triazolam 0.5 mg (40 mg)	Flunitrazepam 2 mg, brotizolam 0.25 mg, diazepam 10 mg, triazolam 0.5 mg (30 mg)	None	None	None (0 mg)	None (0 mg)	Olanzapine 35 mg, quetiapine 25 mg, levomepromazine 25 mg (1462.9 mg)	Olanzapine 35 mg (1400 mg)	16	11	12	9	20	4

Patient characteristics

As shown in Table 2, seven patients discontinued using benzodiazepine hypnotics (discontinuation group), and six had their doses reduced (reduction group).

**Table 2:** Patient demographics and baseline characteristics

Characteristics		Discontinuation group (n=7)	Reduction group (n=6)
Sex, n (%)	Female	4 (57.1)	4 (66.6)
	Male	3 (42.9)	2 (33.3)
Age, years, mean (SD)		51.4 (16.2)	52.2 (10.6)
Benzodiazepine hypnotic, n (%)	Brotizolam	5 (71.4)	0 (0)
	Flunitrazepam	1 (14.3)	6 (100)
	Nitrazepam	1 (14.3)	0 (0)
Outpatient / inpatient, n (%)		4 (57.1) / 2 (42.9)	3 (50) / 3 (50)
Concurrent medical condition, n (%)	Anorexia nervosa	1 (14.3)	0 (0)
	Major depressive disorder	4 (57.1)	1 (16.7)
	Schizophrenia	1 (14.3)	5 (83.3)
	Sleep disorder only	1 (14.3)	0 (0)
Medication, mg, mean (SD)	Diazepam equivalent dose	8.8 (3.4)	16.7 (13.6)
	Imipramine equivalent dose	115.7 (150.6)	12.5 (30.6)
	Chlorpromazine equivalent dose	141.0 (212.2)	611.1 (466.9)
Subjective sleep score, mean (SD)	Mean (SD) ISI score	12.0 (3.7)	8.2 (5.6)
	PSQI total score	10.1 (3.5)	10.3 (2.4)

The discontinued benzodiazepine hypnotics encompassed brotizolam (five patients, 71.4%), flunitrazepam (one patient, 14.3%) and nitrazepam (one patient, 14.3%). The benzodiazepine hypnotics with reduced doses included flunitrazepam, which was used by six patients (100%). The comorbidities were major depressive disorder in four patients (57.1%) in the discontinuation group and schizophrenia in five patients (83.3%) in the reduction group. The imipramine-equivalent dose was 115.7 mg in the discontinuation group and 12.5 mg in the reduction group. The chlorpromazine-equivalent dose was 141.0 mg in the discontinuation group and 611.1 mg in the reduction group.

Change in the use of benzodiazepine and concomitant medications

**Figure 1:**
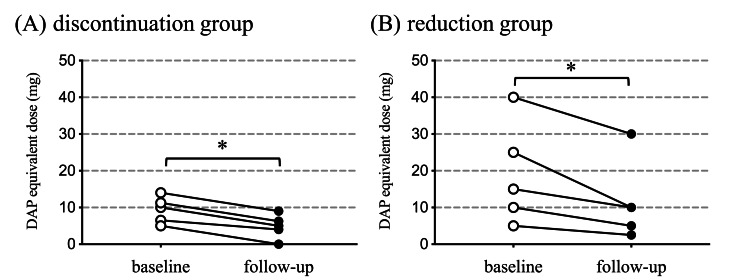
Changes in benzodiazepine dose Comparison of benzodiazepine hypnotic use at baseline and follow-up by the discontinuation (A) and reduction (B) groups. The use of benzodiazepine hypnotics was calculated using the diazepam (DAP)-equivalent dose. p-values reflect the significance of the changes from baseline and were determined using the Wilcoxon matched-pairs signed rank test. (A) p < 0.05, (B) p < 0.05.

The diazepam-equivalent dose significantly decreased after the benzodiazepine hypnotics were discontinued or reduced (Figure 1A-1B).

**Figure 2:**
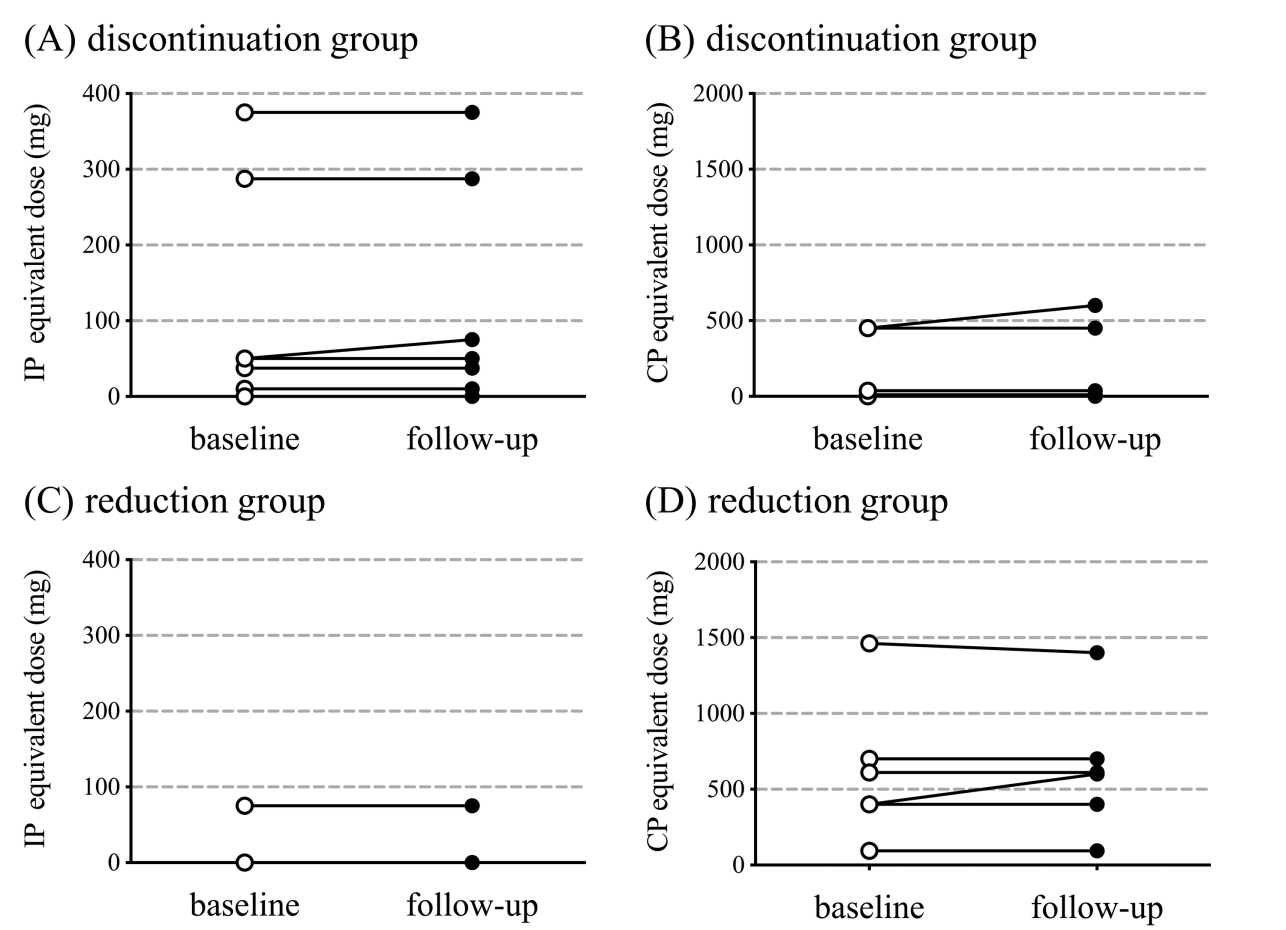
Changes in antidepressant and antipsychotic dose Comparison of use of concomitant drugs at baseline and follow-up by the discontinuation and reduction groups. The dosage of antidepressants was calculated using the imipramine (IP)-equivalent dose, and the dosage of antipsychotics was calculated using the chlorpromazine (CP)-equivalent dose. (A, B) Changes in the IP- and CP-equivalent doses at baseline and during follow-up for the discontinuation group. (C, D) Changes in the IP- and CP-equivalent doses at baseline and during follow-up for the reduction group. P-values reflect the significance of changes from baseline analyzed with the Wilcoxon matched-pairs signed rank test. (A) p > 0.99, (B) p > 0.99, (C) p-value could not be calculated due to similarity, and (D) p > 0.99.

In this study, benzodiazepine hypnotics were either discontinued or reduced, whereas the benzodiazepine anxiolytics remained unchanged. The changes in the diazepam-equivalent dose from baseline were -4.6±0.9 mg (discontinuation group) and -6.7±4.9 mg (reduction group) at follow-up. The equivalent doses of chlorpromazine and imipramine did not change significantly from baseline to follow-up in the discontinuation and reduction groups (Figure 2A-2D).

The mean change in the diazepam (DAP) equivalents for the benzodiazepine hypnotics was 2.9±2.3 mg/week for the reduction group and 1.7±0.6 mg/week for the discontinuation group.

Primary efficacy analysis (assessment of insomnia severity)

**Figure 3:**
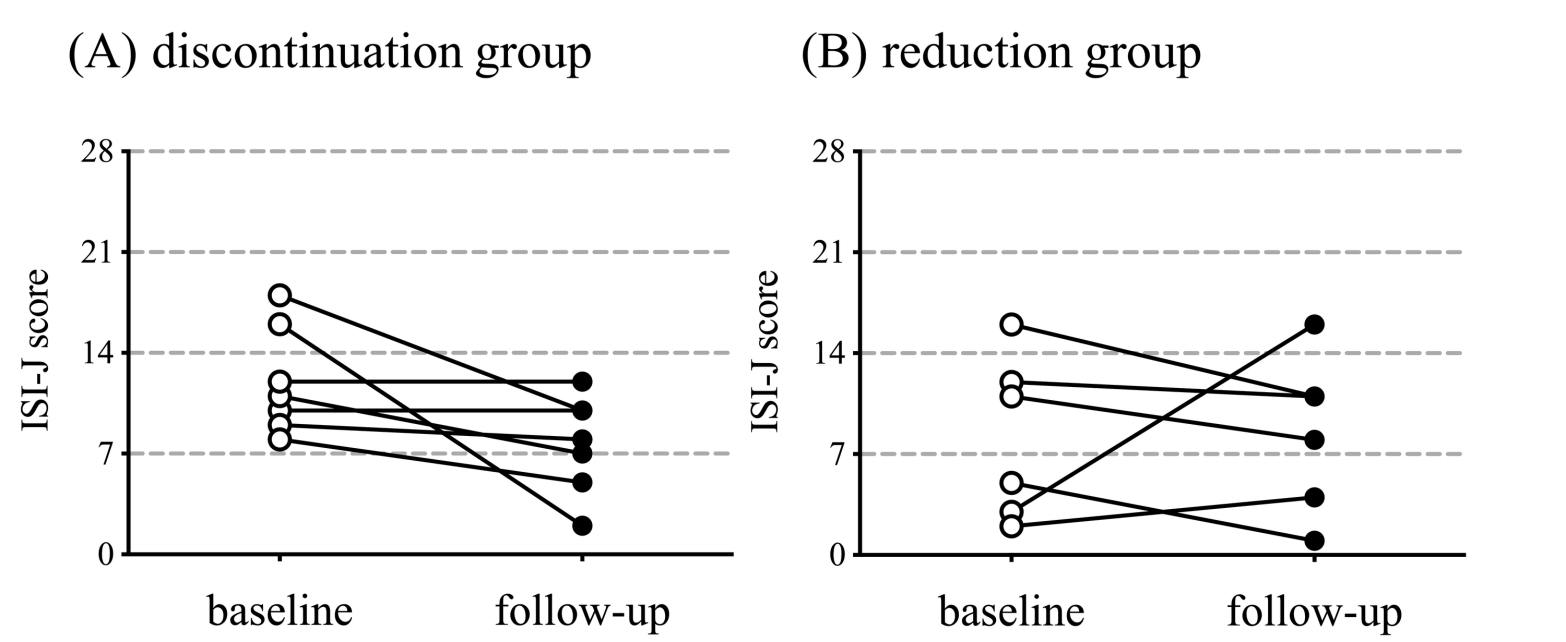
Change in insomnia severity Comparison of the Insomnia Severity Index Japanese version (ISI-J) scores at baseline and during follow-up for the discontinuation (A) and reduction (B) groups. P-values reflect changes from the baseline analyzed using paired t-test. (A) p = 0.07, (B) p = 0.91.

As shown in Figure 3A-3B, the mean rating ISI-J scores, categorized by every 7 points, at baseline were 12.0 ± 4.0 and 8.2 ± 5.6 points for the discontinuation and reduction groups, respectively.

At baseline, five patients had subthreshold insomnia, and two had moderate-severity clinical insomnia in the discontinuation group. Three patients had no clinically significant insomnia, two had subthreshold insomnia, and one had moderate-severity clinical insomnia in the reduction group. Five patients in the discontinuation group had reduced ISI-J scores from baseline; however, the mean ISI-J total score was not significantly reduced (p = 0.07). Similarly, four patients in the reduction group had reduced ISI-J scores from baseline, but the mean ISI-J total score was not significantly reduced (p = 0.91). Of the present patients, 10 (five in the discontinuation group, and five in the reduction group) showed baseline ISI-J scores of less than 14 points.

Secondary efficacy analysis

Assessment of Subjective Sleep Quality and Safety

**Figure 4:**
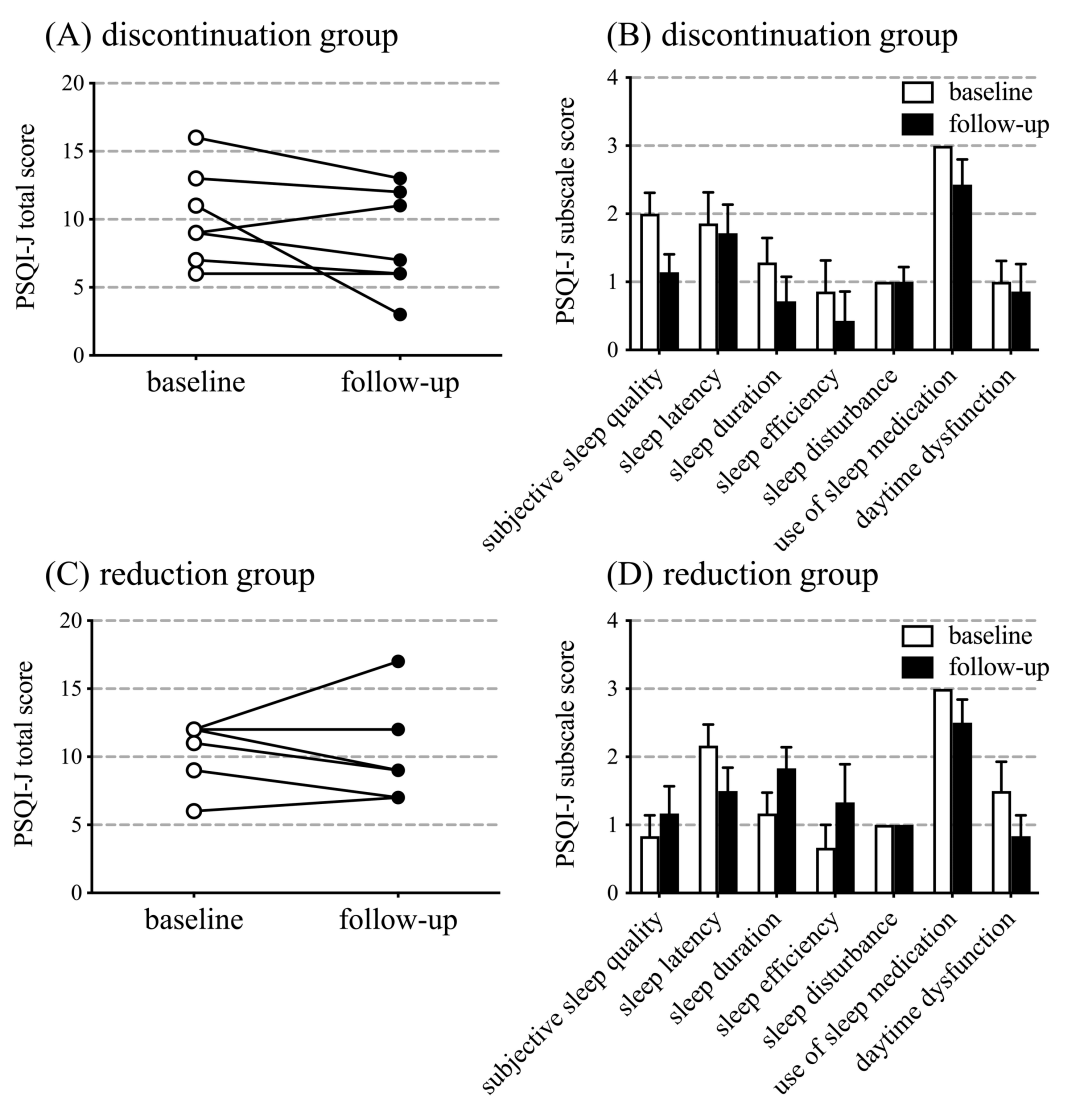
Pittsburgh Sleep Quality Index score Comparison of the total scores of the Pittsburgh Sleep Quality Index Japanese version (PSQI-J) and 7 subscale score of the PSQI-J at baseline and during follow-up for the discontinuation (A, B) and reduction (C, D) groups. P-values reflect the significance of changes from the baseline analyzed using the Wilcoxon matched-pairs signed rank test. (A) p = 0.19; (B) p = 0.13 (subjective sleep quality discontinuation), p > 0.99 (sleep latency), p = 0.50 (sleep duration), p = 0.50 (sleep efficiency), p > 0.99 (sleep disturbance), p = 0.50 (use of sleep medication), and p > 0.99 (daytime dysfunction); (C) p = 0.19; (D) p = 0.75 (subjective sleep quality discontinuation), p = 0.25 (sleep latency), p = 0.25 (sleep duration), p = 0.31 (sleep efficiency), p-value could not be calculated due to similarity (sleep disturbance), p = 0.50 (use of sleep medication), and p = 0.25 (daytime dysfunction).

The PSQI-J scores did not change significantly from baseline to follow-up for the discontinuation and reduction groups (Figure 4A, 4C). All patients had clinical insomnia based on their PSQI-J scores. Approximately seven subscale scores (subjective sleep quality, sleep latency, sleep duration, sleep efficiency, sleep disturbance, use of sleep medication, and daytime dysfunction) for PSQI-J in the discontinuation group, six subscale scores (excluding sleep disturbance) marginally improved from baseline (Figure 4B). In the reduction group, the scores for sleep latency, use of sleep medication, and daytime dysfunction improved from baseline (Figure 4D).

Assessment of Anxiety Symptom Severity

**Figure 5:**
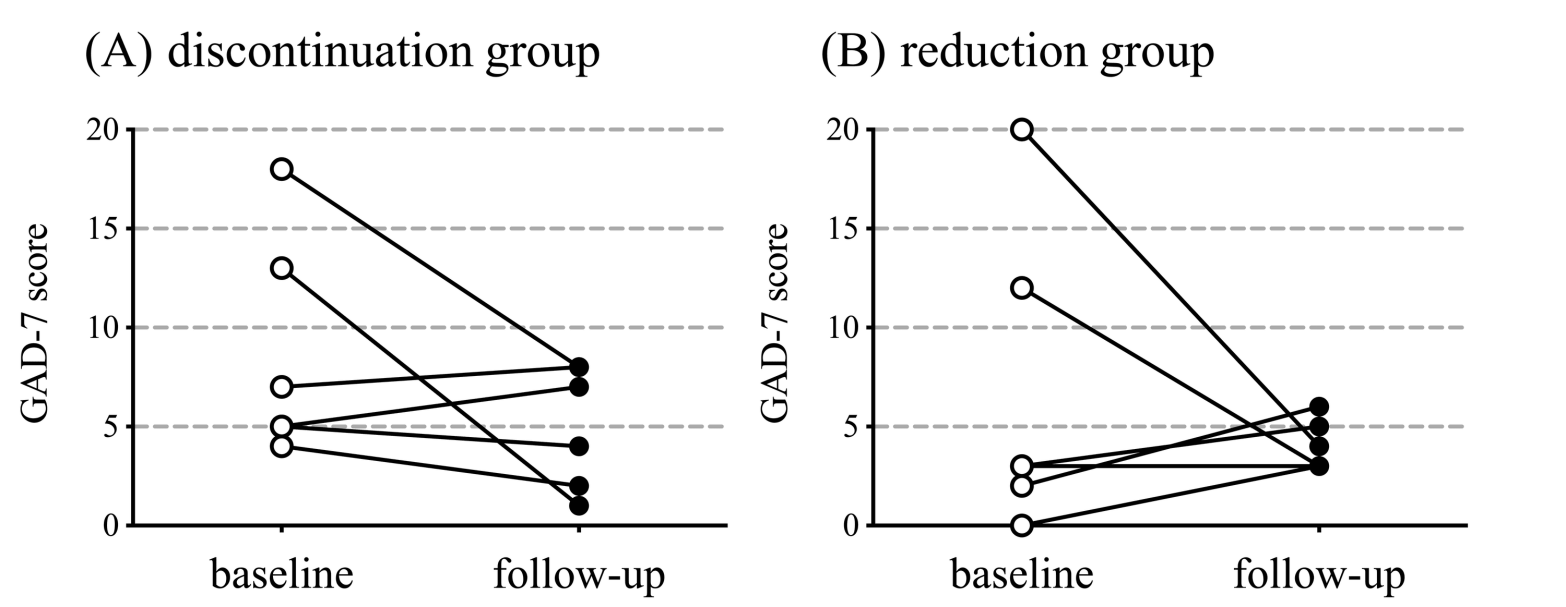
Generalized Anxiety Disorder 7 score Comparison of the Generalized Anxiety Disorder 7-item (GAD-7) total scores at baseline and during follow-up for the discontinuation (A) and reduction (B) groups. P-values reflect the significance of changes from baseline analyzed using the Wilcoxon matched-pairs signed rank test. (A) p = 0.27, (B) p = 0.81.

The GAD-7 scores did not change significantly from baseline to follow-up (Figure 5A-5B).

The GAD-7 scores were stratified at 6-point intervals. None of the patients had an increase in their GAD-7 score of 6 points or more.

Safety

Adverse events with an incidence of >10% over four weeks in each group are summarized in Table 3.

**Table 3:** Summary of adverse events Adverse events occurred in approximately 10% of cases. P-values reflect changes in adverse events throughout the study using Fisher's exact test.

Adverse events		Baseline	During follow-up	p vs. Baseline
Discontinuation group, n (%)	Somnolence	3 (42.9)	3 (42.9)	>0.99
	Headache	2 (28.6)	1 (14.3)	>0.99
	Fatigue	4 (57.1)	3 (42.9)	>0.99
	Irritation	1 (14.3)	3 (42.9)	0.56
	Abnormal dreams	4 (57.1)	3 (42.9)	>0.99
	Dizziness	2 (28.6)	1 (14.3)	>0.99
	Nausea	0 (0)	0 (0)	>0.99
Reduction group, n (%)	Somnolence	3 (50)	4 (66.7)	>0.99
	Headache	1 (16.7)	0 (0)	>0.99
	Fatigue	3 (50)	3 (50)	>0.99
	Irritation	2 (33.3)	2 (33.3)	>0.99
	Abnormal dreams	2 (33.3)	4 (66.7)	0.57
	Dizziness	0 (0)	1 (16.7)	>0.99
	Nausea	0 (0)	1 (16.7)	>0.99

Common adverse events observed in both groups during the study were somnolence, headaches, fatigue, irritation, abnormal dreams, dizziness, and nausea. However, the overall incidence of these adverse events did not differ significantly for the groups during the study relative to the baseline.

Persistence rate

**Figure 6:**
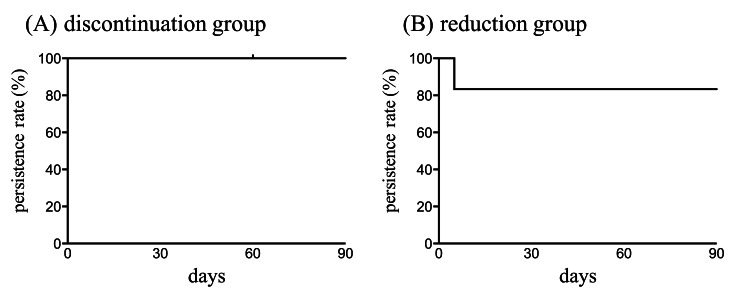
Persistence rate for the discontinuation or dose reduction of the benzodiazepine hypnotics The persistence rate for the discontinuation or dose reduction of the benzodiazepine hypnotics was shown. Baseline was defined as day 0, when benzodiazepine hypnotics were discontinued or reduced. One patient was untraceable on day 60 due to transfer to another hospital.

All patients in the discontinuation group and five patients (83.3%) in the reduction group maintained the changes in their use of hypnotics for three months (Figure 6A-6B).

Only one patient in the reduction group had the dose increased to the baseline because of the worsening of the sleep condition. The ISI-J score worsened from 3 to 16 points, the PSQI-J score worsened from 12 to 17 points, and the GAD-7 score remained unchanged. One patient in the discontinuation group could not be traced on day 60 following a transfer to another hospital.

## Discussion

BZRAs have long served the medical community as primary treatments for insomnia and anxiety symptoms [21]. However, the adverse events resulting from GABAergic neural effects have recently become a social problem [2, 22]. Therefore, short-term use of BZRAs is recommended [5], and dose reduction or discontinuation should be considered for long-term use. However, few studies have examined the effects of reducing or switching BZRAs that have been used for a long time. Evidence is needed to evaluate the effects of dose reduction or discontinuation of these long-term BZRA hypnotics, but since large-scale clinical trials are difficult to conduct, small-scale studies and case reports need to be accumulated. This present study examined the effects of reducing the dose of BZRAs or discontinuing BZRAs hypnotics after use for more than 12 months on sleep conditions and anxiety symptoms.

This study showed that sleep conditions and anxiety symptoms did not worsen when the dose of BZRA hypnotics was reduced or patients were switched to other hypnotics. Generally, dependence is reported to develop after eight months or more of the use of BZRA hypnotics [9, 13], and the patients in this study were considered to have been using BZRA hypnotics for a duration sufficient for dependence to develop. However, in the present case, the risk of withdrawal symptoms was low because the dose reduction was less than 25% per week of the DAP equivalent [22]. This may explain why the reduction and switch were safe, except for one case in which symptoms characteristic of withdrawal developed. In addition, 10 of the present patients (78%) showed baseline ISI-J scores of less than 14 points, indicating that their cases of insomnia were clinically mild and could be considered well-controlled with the current treatment. This may contribute to the success of reducing or switching BZRA hypnotics in the long term. Based on the results of this study, it may be worthwhile to attempt dose reduction in patients who have been using BZRA sleep medications for a long time, if they have good sleep control.

However, the influence of concomitant medications should also be considered. In the present study, the participants were patients with psychiatric disorders who were taking antipsychotic and/or antidepressant medications. In particular, the antipsychotic quetiapine [23] and antidepressant trazodone [24, 25] are known to have hypnotic effects and may have influenced sleep conditions and anxiety symptoms in this study. However, there was no difference in the mean prescribed doses of antipsychotics and antidepressants at baseline and follow-up, with only two patients increasing their dose of antipsychotics and one patient increasing their dose of antidepressants. Therefore, concomitant antipsychotic and antidepressant medications were not considered to have significantly impacted the results of this study.

Withdrawal symptoms and adverse events should also be considered when reducing or switching to BZRA sleep medications. Typical withdrawal symptoms include anxiety, sleep disturbance, and nervousness [26]. However, only one patient showed these symptoms after reducing or switching to BZRA sleeping pills in this study, and a dose reduction or switch was safe and successful for most patients. A possible reason for this is the use of the recommended low-dose titration regimen. One patient who experienced withdrawal symptoms had a 50% dose reduction within a short period, although 10-25% dose reductions of BZRA every two weeks have been reported [5]. Careful monitoring of the rate of dose reduction is recommended. With appropriate approaches, the dose can be reduced without withdrawal symptoms or other adverse events.

Furthermore, switching from benzodiazepine hypnotics to orexin receptor antagonists, such as suvorexant [27] and lemborexant [27, 28], has also been reported. Orexin receptor antagonists have a completely different mechanism of action other than BZRAs, inducing hypnotic effects by inhibiting orexin receptors, which are responsible for promoting arousal. These orexin receptor antagonists may be effective if there is concern that reducing the doses of or discontinuing benzodiazepine hypnotics may worsen sleep.

The limitations of this study were as follows. The first is the study design and scale. This was a single-center retrospective study. Therefore, patients from various backgrounds were included in the study because their ages and primary illnesses could not be standardized. Further studies with large sample sizes and objective sleep measures are necessary to validate and expand upon these findings. Second, sleep conditions were analyzed using a self-reporting questionnaire. In sleep evaluation, objective assessments such as polysomnography [29] and actigraphy [30] are generally required. However, these objective assessments are difficult to perform in daily clinical practice and data could not be obtained in this retrospective study. Third, the psychiatrists determined whether the BZRA hypnotics, which had been used over the long term, could be reduced or switched in all patients. Furthermore, most of the patients had mild insomnia. Therefore, it may have been easier to reduce the dose of the BZRA hypnotics for them. Fourth, there might be a bias in hypnotics, comorbidities, and concomitant medications that were discontinued/reduced in the discontinuation and reduction groups. The effects of these biases could not be compared in detail due to the small sample size.

The strength of this study is that it highlights changes in both sleep and anxiety associated with the reduction or discontinuation of benzodiazepine hypnotics, which have not been previously reported. This study might encourage reconsideration of long-term benzodiazepine hypnotic use and support efforts to reduce dosage where appropriate.

BZRA hypnotics can potentially be safely switched or their doses can be reduced using appropriate approaches. It is important to attempt dose reduction in patients with good sleep control.

## Conclusions

BZRA hypnotics can be discontinued or their dose reduced after long-term use without worsening sleep conditions and anxiety symptoms. Our results may provide a basis for the safe and effective discontinuation or dose reduction of long-term benzodiazepine hypnotics use.
